# P11 Evaluation of *in vitro* activity of double β-lactam ‘therapy’ in *Escherichia coli*: what is the association between ‘synergy’ and *in vitro* susceptibility?

**DOI:** 10.1093/jacamr/dlad066.015

**Published:** 2023-06-14

**Authors:** J Suich, M Van Der Woude, D Wearmouth, P Burns, G Barlow

**Affiliations:** Hull Teaching Hospitals, Hull, UK; York University, York, UK; Hull Teaching Hospitals, Hull, UK; Hull Teaching Hospitals, Hull, UK; Hull Teaching Hospitals, Hull, UK

## Abstract

**Background:**

Emerging antimicrobial resistance is a leading cause of patient morbidity/mortality worldwide. A potential strategy in the treatment of resistant infections is the use of combination antibiotics, with the hope of achieving greater *in vitro* activity, including synergy. It is currently assumed that combinations generating the greatest synergistic effect *in vitro* will be associated with the greatest improvement in *in vitro* susceptibility (as defined by EUCAST—see Methods). The aim of this study was to determine if this was indeed the case.

**Methods:**

We systematically determined the relationship between double β-lactam therapy against 7 *Escherichia coli* strains of variable resistance *in vitro* (4 clinical/3 purchased). This included fully susceptible and low-level resistance isolates (*N* = 4), extended-spectrum β-lactamase producers (ESBLs) (*N* = 2) and carbapenemase producers (CPEs) (*N* = 1). For each of 10 β-lactam antibiotics, the MIC was determined individually, and subsequently in combination, using the MTS^™^ ‘cross’ synergy method (Liofilchem, 2012). The fractional inhibitory concentration (FIC) was calculated by dividing the MIC of each drug in combination by the MIC of each drug alone and adding the results. See Figure 1. Results were analysed according to EUCAST clinical breakpoints to determine if synergistic combinations correlated with a potentially improved *in vitro* susceptibility phenotype. A susceptibility phenotype improvement was defined: R/R or R/I or I/I monotherapy, improving to R/S, I/S or S/S combination therapy (where S = susceptible, I = intermediate and R = resistant).

**Results:**

Overall, 86/630 (13.7%) combinations showed synergy; 408/630 (64.7%) = additive; 136/630 (21.6%) = indifferent. No antagonism was identified. Synergy was most detected in ESBL producers (32% of combinations; Figure 2) and less frequently in CPEs (2% combinations) and fully susceptible isolates (4% combinations). 31/630 (5%) combinations showed improvement in EUCAST susceptibility between mono/combination therapy (according to our definitions). Of these 31 combinations, 8 (26%) were synergistic, 15 (48%) additive and 8 (26%) indifferent (by FIC index). Of 599/630 combinations that showed no improvement in susceptibility, 78 (13%) = synergistic, 393 (65.6%) = additive and 128 (21.4%) = indifferent. It is however important to note that in 284/630 (45%), initial monotherapy susceptibility was S/S (i.e. further improvement not possible). Synergy was more common in combinations that improved EUCAST susceptibility versus those that did not; χ^2^, *P* = 0.04). Ceftazidime/avibactam + amoxicillin was the combination most commonly associated with synergy (12/86 synergistic combinations).

**Conclusions:**

In the β-lactam combinations tested, synergy or additive effects were common (78.4%), similar to our previous work with Fosfomycin/β-Lactam combinations (89%), but higher than fosfomycin/non-β-lactam combinations (28%). Synergy was most detected in ESBL producers with most synergistic combinations containing one/two β-lactamase inhibitors. Overall, however, this rarely leads to an improvement in *in vitro* susceptibility by EUCAST criteria and is highly bug–drug–drug combination dependent. Further work using alternative validating methodology and in more isolates is required.

**Figure 1.**
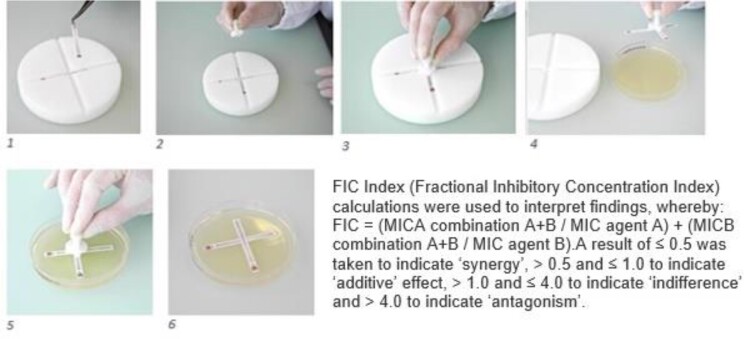
MTS^™^ ‘cross’ synergy method (Liofilchem, 2012).

**Figure 2.**
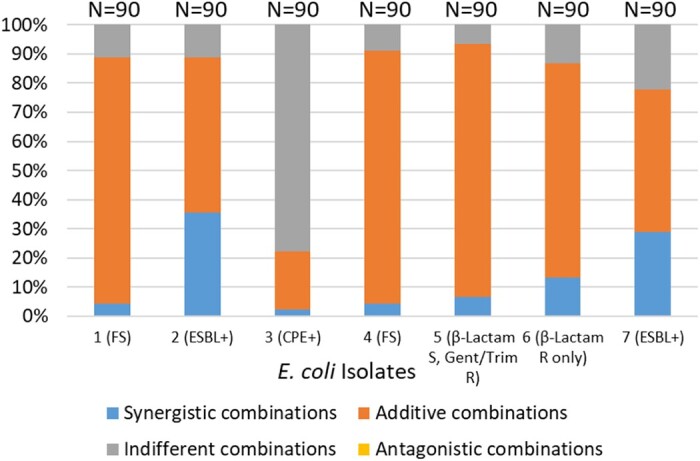
β-Lactam antibiotics in combination versus 7 *E. coli* isolates of variable resistance *in vitro*.

